# On the Absence of Typhus in the Tropics, and in the Southern Half of the Earth

**Published:** 1855-07

**Authors:** 


On the Absence of Typhus in the Tropics, and in the Southern Half of the Earth. By Dr. M——. In an interesting paper, this anonymous writer passes in review the various evidences found in writers which show that the European typhus is unknown, or almost so, in the tropics and in the Southern Hemisphere. He appears to be extremely well read in English literature, and draws many of his illustrations from English authors. He has not even omitted the late observations on the occurrence of three cases of typhoid fever in Burmah, by Mr. Scriven;* but he considers these to be doubtful. His conclusion is, that "Typhus is a disease only of the Northern temperate zone, and that its Southern limit is the isothermal line of 72° Fah.; it does not occur in the Southern temperate zone, or at any rate it is not endemic there."—*Ibid.*

---

* Medical Times and Gazette, Feb. 1854.

